# Epstein-Barr virus: From kisses to cancer, an ingenious immune evader

**DOI:** 10.18632/oncotarget.26381

**Published:** 2018-11-23

**Authors:** Pankaj Trivedi, Frank J. Slack, Eleni Anastasiadou

**Affiliations:** Harvard Medical School Initiative for RNA Medicine, BIDMC, Harvard Medical School, Boston, MA, USA; Department of Experimental Medicine, Sapienza University of Rome, Rome, Italy

**Keywords:** EBV, PD-L1, microRNA, immune checkpoints, lym-phoma

A long battle, which is still raging, is between viruses and the host immune system. Generally, the very efficient control of infectious agents by the immune system either eradicates the invaders or constrains the "guests" to a more or less peaceful coexistence, like in the case of latent herpesvirus infections. The latter arrangement however, comes into effect only after a virus has successfully avoided the initial immune onslaught by utilizing ingenious methods. In this regard, Epstein-Barr virus (EBV) could be considered one the most successful parasites, because in spite of its notorious ability to transform B cells, it has managed to infect over 90% of the human population. It is forced to adopt a benign nature by a vigilant immune system. In fact, in immunocompromised individuals such as transplant or HIV infected patients, the virus can create havoc as most lymphomas in such patients are caused by EBV.

EBV is associated with a wide variety of human cancers [1, 2] and also autoimmune diseases [3]. In a healthy immunocompetent host, in order to establish latency, EBV downregulates its immunogenic proteins and gains access to the memory B cell compartment. In pathological conditions, like in Burkitt lymphoma (BL), the virus can downregulate HLA molecules, influence peptide transport associated protein (TAP) expression, change its immunogenic epitopes when a given MHC haplotype is more frequent, and make the only viral protein required for the viral maintenance, EBNA1, non-immunogenic [4] [5].

Immune checkpoints (IC) contain a set of proteins, which controls T cell responses so that our body is not harmed by the very cells, which defend us. The IC proteins deliver two opposing signals to T cells, namely: Attack or Do not attack. We can compare these proteins to switch on/switch off signals to T cell responses. Evolution selected for these mechanisms to avoid autoimmune diseases and to ensure that there is no collateral damage while T cells are working to neutralize invading agents [6]. Cancer cells usurp these inherent IC controls to their own ends by either increasing T cell inhibitory proteins or decreasing the T cell stimulatory protein quantity on their cell surface to cause T cell exhaustion and compromise anti-tumor immunity [7].

Most cancers occur in people with functioning immune defenses. Therefore, as mentioned before, to survive, thrive and be evolutionary successful, viruses have devised strategies to avoid host immune responses [4]. One of these strategies may involve either viral or cellular miRNAs, the non-coding post-transcriptional gene regulators, to influence IC proteins. In order to find efficient therapy of virally associated cancers, it is essential that we understand how viruses alter miRNAs to subvert the immune system [8].

EBV infected cancers have higher expression of PD-L1 but the precise involvement of virally encoded proteins and molecular mechanisms behind its increased expression was not known. In a recent paper Anastasiadou et al., describe a trick used by EBV to evade immune surveillance [9]. Through its most transforming protein, EBNA2, it induces PD-L1 expression and makes the infected cancer cell invisible to T cells. A series of EBV infected lymphoma cell lines and EBNA2 transfected derivatives were tested for PD-L1 upregulation. Interestingly, EBNA2 but not LMP1 upregulated PD-L1.

Since miRNAs play a critical role in post-transcriptional gene regulation [10, 11], we investigated if PD-L1 regulating miRNAs are downregulated in EBNA2 expressing DLBCL cells. miR-34a is a well-known tumor suppressor miRNA and is regulated by p53. This miRNA regulates PD-L1 [12]. First through microarrays and subsequently by real time qPCR, it was observed that miR-34a expression is strongly reduced in EBNA2 expressing lymphoma cells. When miR-34a mimics were reintroduced in lymphoma cells expressing EBNA2, PD-L1 went down. This and reporter assays with PD-L1 3' UTR confirmed that it is indeed an authentic miR-34a target. In addition, pre-miR-34a was underexpressed in EBNA2 transfectants and this suggests that the viral protein is affecting miR-34a transcription.

To identify molecular mechanisms behind miR-34a downregulation by EBNA2, we resorted to EBNA2 CHIP-seq datasets in GEO database. We found that EBNA2 peaks at the miR-34a promoter. JASPER database and Integrative Genomic viewer helped us identify that at least one consensus binding site of EBF1 at miR-34a promoter coincides with the EBNA2 peak. We reasoned that EBNA2 might recruit EBF1 to miR-34a promoter and repress it. Indeed, introduction of lentiviral vectors with shEBF1 led to derepression of miR-34a and downregulation of PD-L1. A novel repressor function of EBF1 on miR-34a transcription was thus identified.

The immunological significance of EBNA2-PD-L1-miR-34a connection, was studied with 3D microfluidic chips. The EBNA2 transfected DLBCLs were poorly immunogenic and IFN-gamma production was hampered. When miR-34a was reconstituted in EBNA2 transfectants, IFN-gamma production was boosted in both CD4 and CD8 T cells. In the first ever use of a 3D biomimetic chip to evaluate tumor immunogenicity, miR-34a transduction in EBNA2 expressing lymphoma cells reconstituted their immunogenic potential.

The *in vitro* data are quite clear and conclusive but what about clinical samples from lymphoma patients? In a small cohort of DLBCL biopsies, it was observed that EBV positive DLBCLs have higher PD-L1 expression in comparison to EBV negative ABC DLBCLs. The staining intensity and number of PD-L1 positive cells was further increased when the samples were EBNA2 positive. This was the definitive proof that EBNA2, through inducing PD-L1, may compromise tumor specific T cell responses and thus may contribute to poorer prognosis of EBV+/EBNA2+ DLBCLs.

In the light of these findings, a few issues need further consideration. The foremost is that EBNA2 is not frequently expressed in most EBV carrying tumors. This however, does not rule out that in the course of the tumor development, it was never expressed (Lat IIb). It is also conceivable that in EBNA2 negative tumors, other EBV encoded proteins might be delegated with the task of ensuring high PD-L1. Indeed, it was recently shown that lymphomas in EBNA1 transgenic mice are high PD-L1 expressors [13]. Furthermore, whether EBV affects other immune checkpoints remains to be determined. Ideally, confirmation of our findings in a larger EBNA2 positive DLBCL cohort will be important. But overall, based on our data, we propose that EBV positive DLBCLs could be a suitable category of lymphomas for clinical trials based on immune checkpoint inhibitors.

**Figure 1:**
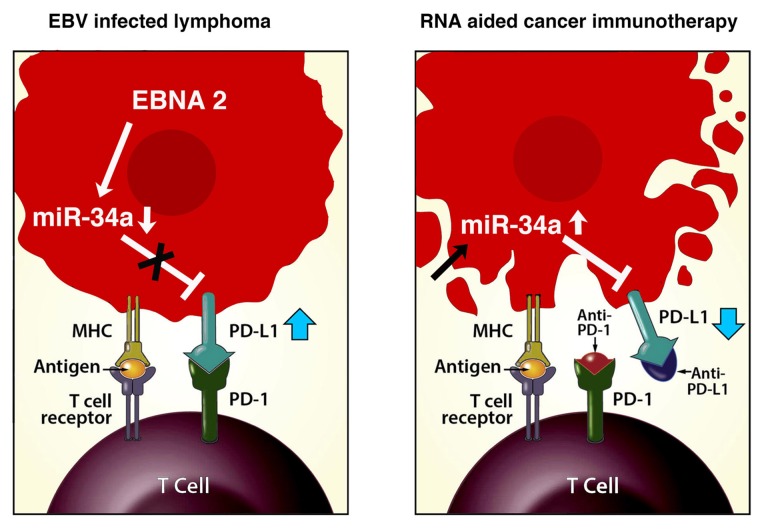
A model for RNA aided cancer immunotherapy The panel on the left summarizes how EBV encoded EBNA2 downregulates miR-34a and increases PD-L1 expression. The right panel shows miR-34a and immune checkpoints inhibitors based combinatorial approach to improve efficacy of cancer immunotherapy.

As the protracted war between invading pathogens and the immune system goes on and each combatant is busy deploying new strategies and countermeasures, we urgently need to help cancer patients. The 3D biomimetic chips could be very useful in rapid evaluation of novel compounds to reconstitute tumor immunogenicity. Cancer immunotherapy has been very effective but unfortunately only in a small number of patients. The immune system needs to be fortified to clear the virus and together with it also cancer. RNA aided immunotherapy (Figure 1) could provide that helping hand.
